# Kinesin spindle protein inhibitors in cancer: from high throughput screening to novel therapeutic strategies

**DOI:** 10.2144/fsoa-2021-0116

**Published:** 2022-02-21

**Authors:** Rand Shahin, Salah Aljamal

**Affiliations:** 1 he Hashemite University, Faculty of Pharmaceutical Sciences, Department of Pharmaceutical Chemistry, P.O. Box 330127, Al-Zarqa 13133, Jordan; 2 Faculty of pharmacy, University of Jordan, Amman, 11942, Jordan

**Keywords:** Eg5, filanesib, ispinesib, *KIF11*, kinesin spindle protein, KSP, litronesib, monastrol

## Abstract

Bringing to a halt the cell cycle in mitosis and interfering with its normal progression is one of the most successful anti-cancer strategies used nowadays. Classically, several kinds of anti-cancer drugs like taxanes and vinca alkaloids directly inhibit microtubules during cell division. These drugs exhibit serious side effects, most importantly, severe peripheral neuropathies. Alternatively, KSP inhibitors are grasping a lot of research attention as less toxic mitotic inhibitors. In this review, we track the medicinal chemistry developmental stages of KSP inhibitors. Moreover, we address the challenges that are faced during the development of KSP inhibitor therapy for cancer and future insights for the latest advances in research that are directed to find active KSP inhibitor drugs.

## Plain language summary

Scientists have recognized the importance of selective KSP inhibitors in the early 2000s and so various KSP protein inhibitors have been investigated. Only ten of these have been clinically evaluated for cancer treatment. Ispinesib (SB-715992) and filanesib (Arry-520) were the most promising small molecules in clinical trials against the KSP protein. Many challenges are faced during the development of an active anti-KSP drug; most importantly are the unsatisfactory clinical trial results. Designing dual inhibitors, antibody–drug conjugates, combination therapy and gene therapy approach are among the main strategies that are being investigated nowadays to find new effective KSP inhibitors. The scientific research efforts are still devoted to find an effective and tolerable KSP inhibitor drug that can gain US FDA approval.

## Graphical abstract

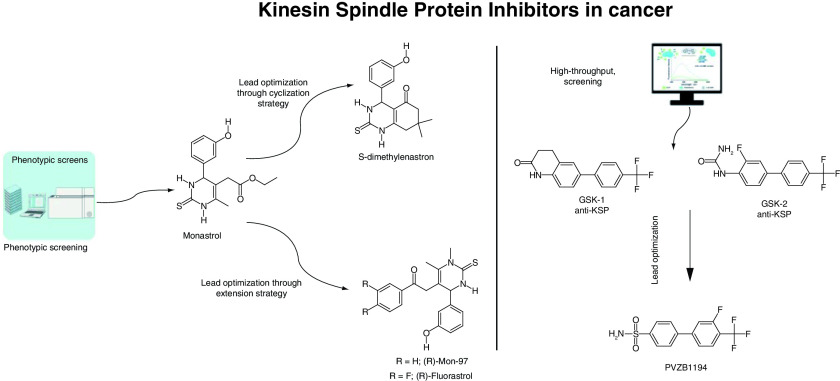

KSP, also known as Eg5, is a member of the kinesin motor superfamily. This superfamily of molecular motors utilizes the energy of hydrolyzed ATP to transport moving organelles inside the cell such as vesicles and microtubules. Inhibition of KSP activity arrests cells in metaphase by forming the characteristic phenotype of aberrant monopolar spindles also named mono astral [1,2]. The mono astral phenotype results from impaired centrosomal separation. The Inhibition of KSP leads to stopping the mitosis process in the target cell without directly disturbing the microtubules. All of this makes KSP an interesting target for drug design in cancer chemotherapy.

Furthermore, the activated KSP-coding gene *KIF11* was detected in relapsed neuroblastoma oncogenic signaling pathways and many other cancer conditions [3]. This also sparked the researchers to intensify their efforts toward finding new KSP inhibitors in order to treat children with high-risk neuroblastoma and prevent their relapse [3].

The KSP protein is composed of 1057 amino acids with three main domains: the motor domain, the stalk domain and the tail domain [1,4]. The motor domain is responsible for hydrolyzing the ATP and generating the energy that is required for moving the microtubular fibers. Meanwhile, the stalk and the tail domains are responsible for dimerization and interaction. Overall, KSP inhibition will lead to cell cycle arrest and apoptosis [5].

Additionally, the ATP binding pocket within the motor domain is called the P loop. Another specific allosteric site within the KSP motor domain is the helix α2/loop L5 and the helix α3 abbreviated as α2/loop L5/helix α3 domain which is almost 12 Å away from the ATP binding site [4,5]. Moreover, a third inhibitory area is also detected in the KSP motor domain named helix-α4 and -α6 pocket [4].

The scientists have recognized the importance of highly selective and targeted KSP inhibitors in the early 2000s [4,6–8]. Since then, there have been many research trials to find anew anticancer drug that targets the KSP enzyme. Unfortunately, the clinical efficacy of the new anti-KSP small molecules has been always a burning issue since the potential KSP inhibitors that used to show relatively good efficacy *in vitro* have demonstrated little or even no antitumor activity *in vivo*.

Various chemical scaffolds were developed as KSP inhibitors including the quinazolinone, the dihydropyrimidines and the thiadiazole derivatives. According to the published crystallographic studies, some of the KSP inhibitors bind to the loop 5 binding allosteric site named the α2/loop L5 helix α3 region and others bind to the helix-α4 and -α6 pocket [4]. Actually, it was found that the 4-aryl-3,4-dihydropyrimidin-2(1H)-ones derivatives such as monastrol, Enastron, fluorastrol, MK-0731 in addition to S-trityl-lcysteine (STLC), ispinesib (SB-715992, CK0238273), litronesib (LY2523355), and filanesib (Arry-520) bind to the α2/loop L5/helix α3 region. Meanwhile, the biaryl compounds such as GSK-1 and GSK-2 in addition to PVZB1194 bind at the junction of helix-α4 and -α6 pocket [4,7].

The previously mentioned KSP inhibitors represent the second generation of anti-mitotic drugs. On the other hand, the first-generation anti-mitotic drugs such as taxanes and epothilones which are microtubule stabilizers in addition to vinca alkaloid which is a microtubule de-stabilizer are associated with many toxicities and resistance issues [9]. Second-generation KSP inhibitors are designed to overcome the acquired resistance and the mechanism-based toxicities of the traditional first-generation anti-mitotic agents [10,11].

In this review, we present the investigational, preclinical and clinical developmental stages of the most important KSP inhibitors. Ten KSP inhibitors are being clinically investigated as potential anticancer drugs. More specifically, 45 phase I/II trials against several kinds of cancer disease have been completed or terminated [12]. In this context, we address the challenges that are faced during the development of KSP inhibitor therapy and the future insights for the latest advances in research field that are directed to overcome these challenges.

## KSP inhibitors chemical classes

Since the discovery of S-trityl-L cysteine (STLC) in 1992 as antimitotic agent many KSP (Eg5) inhibitors with various chemical scaffolds have been identified. The initial identification of KSP inhibitors was mainly through a cytotoxicity-based high throughput screening programs that have been carried out by the National Cancer Institute (NCI). Later on, crystallographic studies revealed even more KSP inhibitors.

KSP inhibitors are sub-divided into two main groups according to their mechanism of interaction within the KSP protein:

1. KSP (α2/loop L5/helix α3) inhibitors also named ATP uncompetitive
2. KSP (helix-α4 and -α6 pocket) inhibitors also named ATP binding competitive inhibitors

### KSP (α2/loop L5/helix α3) binding allosteric inhibitors

#### 4-aryl-3,4-dihydropyrimidin-2(1H)-ones derivatives

**Figure 1.**
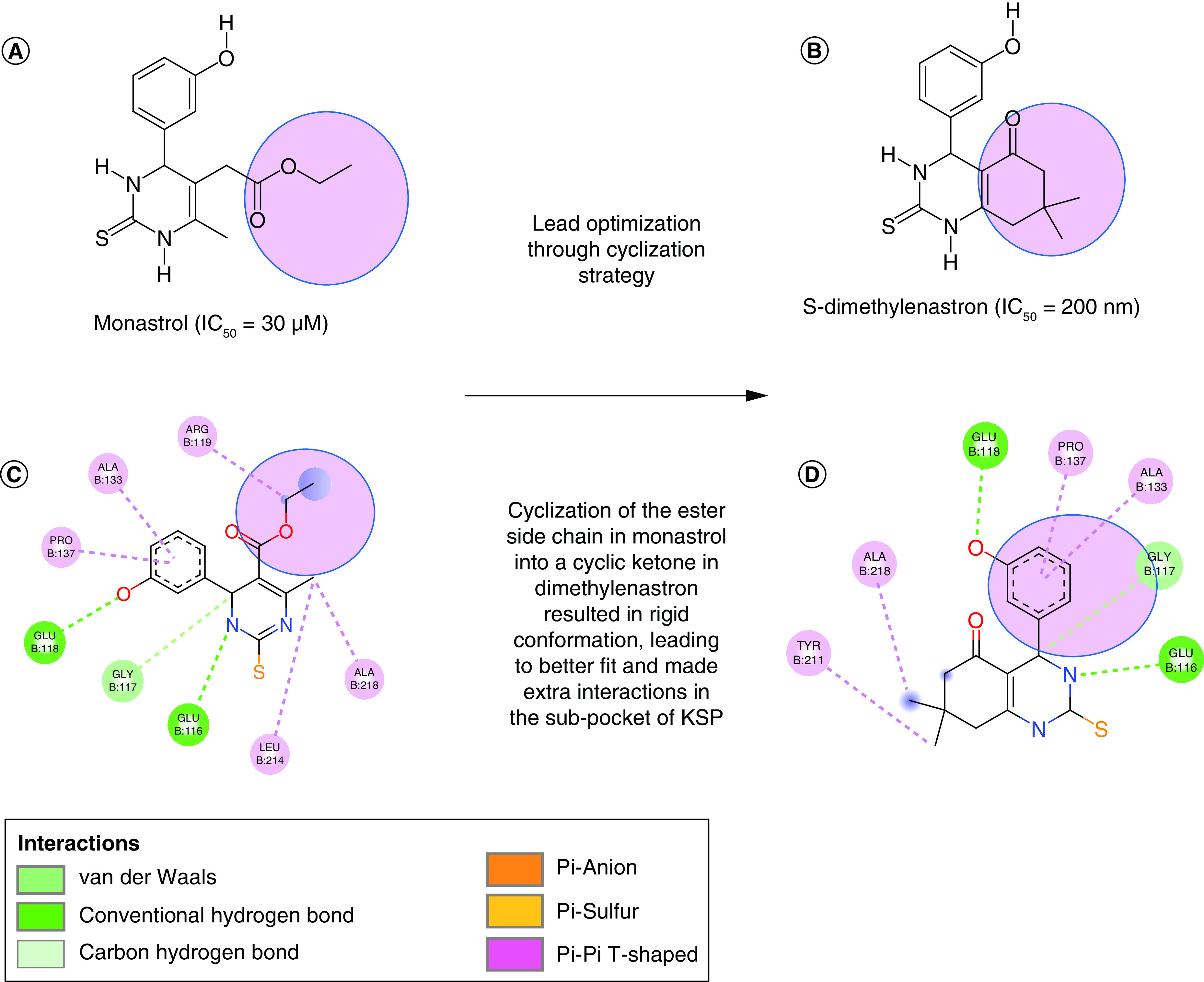
Hit-to lead optimization progress of different class I dihydropyrimidines (DHPM-I) KSP inhibitor chemotypes. **(A)** Structure of the KSP inhibitor monastrol, discovered in 1999. **(B)** Structure of the KSP inhibitor, S-dimethylenastron. **(C)** 2D Structure of monoastral docked inside the KSP binding pocket into KSP crystal (PDB code: 1Q0B, 1.8 Ǻ). **(D)** 2D Structure of S-dimethylenastron docked inside the KSP binding pocket into KSP crystal (PDB code: 2X7D, 2.3 Ǻ). Interactions viewed by Discovery studio visualizer 2021.

The early studies of KSP crystal structures in 1999 identified dihydropyrimidine (DHPM)-derived inhibitors as a novel KSP protein inhibitor (Figure 1A). monastrol was the first to be discovered by Mayer *et al.* in 1999 by a phenotypic screening approach [13]. Mayer's crystallographic research studies confirmed that monastrol inhibits the KSP enzyme by blocking the allosteric site α2/loop L5/helix α3 located almost 12 Å away from the ATP binding pocket leading to mitotic arrest [4].

After binding to the α2/loop L5/helix α3 region, monastrol causes huge conformational changes. These changes prevent adenosine diphosphate (ADP) release which prevented the completion of the catalytic cycle and lead to conformational changes in the microtubules [14]. The main interactions of monastrol inside the KSP binding pocket include the alkyl interactions with Arg119, Ala133, Pro137, Leu214 and Ala218 in addition to the hydrogen bonding interactions with Glu116 and Glu118 (Figure 1C) [1]. Unfortunately, these interactions were not enough to produce a potent and active KSP inhibitor (IC_50_ activity of monastrol was 30 uM against KSP ATPase and 12.3–49.9 µg/ml as cellular potency) [1,15].

Nevertheless, the weak activity of monastrol and its non-drug-like properties prompted the researchers to modify its structure through further lead optimization studies in order to find new KSP inhibitors with better cellular potencies and lesser side effects.

The preliminary structure–activity relationship (SAR) studies focusing on varying the substituents at R1 and R2 identified new DHPM analogs such as enastron and dimethylenastron (Figures 1A–D).

Ester side-chain cyclization of the monastrol into a cyclic ketone in dimethylenastron restricted the number of possible conformations and resulted in optimal rigid conformation, this led to better fit inside the α2/loop L5/helix α3 binding pocket through extra interactions with the amino acid Tyr211 (Figure 1D) [7].

Enastron demonstrated an activity of (IC_50_ = 2μM) against KSP ATPase and dimethylenastron showed an inhibitory activity of (IC_50_ = 200 nM) against KSP ATPase (Figure 1) [16]. The active form of these compounds was the S-enantiomer thus S-enastron, S-dimethylenastron. These compounds were categorized as the class I of DHPM inhibitors that bind in the S configuration to the KSP (Eg5) binding site similar to monastrol [17].

**Figure 2.**
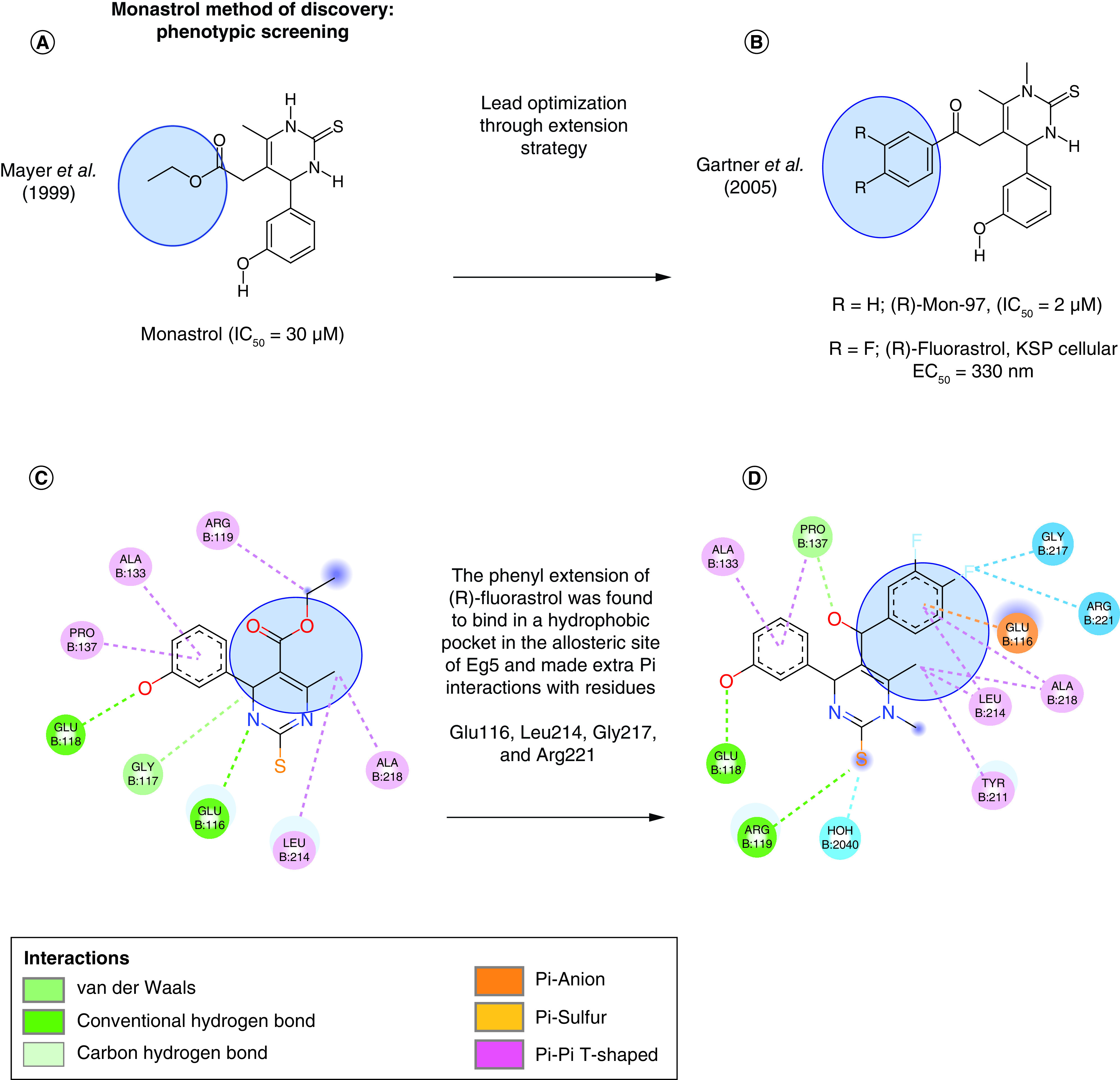
Hit-to lead optimization progress of different class I dihydropyrimidines (DHPM-II) KSP inhibitor chemotypes. **(A)** Structure of the KSP inhibitor, monastrol discovered in 1999. **(B)** Structure of the KSP inhibitor (R)-Mon-97 and (R)-fluorastrol discovered in 2005. **(C)** 2D Structure of monoastral docked inside the KSP binding pocket into KSP crystal (PDB code: 1Q0B, 1.8 Ǻ). **(D)** 2D Structure of (R)-fluorastrol docked inside the KSP binding pocket into KSP crystal (PDB code: 2X7E, 2.4 Ǻ). Interactions viewed by Discovery studio visualizer 2021.

Further SAR studies on dihydropyrimidine (DHPM)-derived inhibitors revealed class II DHPM inhibitors that bind preferentially in the R configuration. Class II DHPM of KSP inhibitors includes mon-97 and fluorastrol (Figure 2).

Fluorastrol is structurally characterized by the two extra fluorine atoms that are attached to the phenyl ring in the Meta- and para-positions (Figure 2B, 2D). Fluorastrol is about five-fold more active than mon-97 when comparing the racemic mixtures and the more active enantiomers. The phenyl extension of (R)-fluorastrol was found to bind in a hydrophobic pocket in the allosteric site of KSP and made extra Pi interactions with residues Glu116, Leu214, Gly217 and Arg221 (Figure 2D).

Class I and II DHMP inhibitor showed two different interaction patterns that were subjected to further optimization steps but unfortunately, the compounds of the dihydropyrimidine group failed to reach the clinical trials because of their lack of efficacy.

#### S-trityl-L cysteine & related compounds

In 1992, Paull *et al.* reported S-trityl-L-cysteine as a new antimitotic agent that inhibits mitosis at the tubulin level [18]. The compound was first discovered as an antimitotic agent through a National Cancer Institute drug evaluation program based on screening various kinds of compounds against 60 human tumor cell lines (GI_50_ value of 1.3 uM).

In 2004, Brier *et al.* reported that S-trityl-L-cysteine specifically binds to the human kinesin KSP through the α2/loop L5/helix α3 binding pocket [19]. STLC causes great structural modification in the neck-linker region and inhibits the KSP function. STLC also shows better anticancer activity against docetaxel resistant prostate cancer cells when compared with monastrol or terpendole E [19].

**Figure 3.**
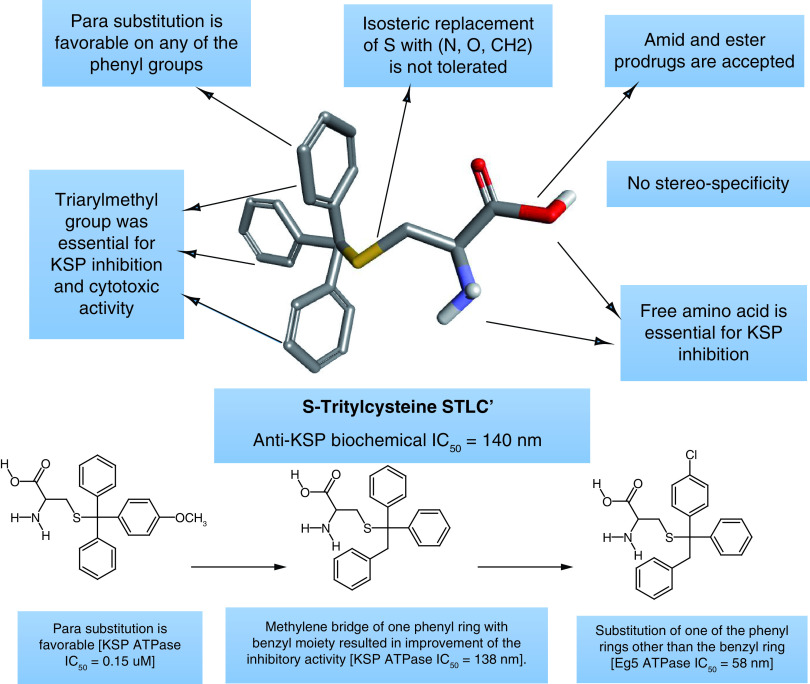
Structure–activity relationship of S-Tritylcysteine STLC as KSP inhibitor.

The use of STLC was limited because of its poor physicochemical properties and reduced cellular permeability. Several modifications were applied to the STLC structure over the years. The amino acid and the tri-phenyl structure of this compound cannot be modified, an extra parasubstitution on the phenyl groups and the prodrug strategy are accepted, please refer to Figure 3 for more details about the STLC structure–activity relationship [7]. None of the STLC derivatives has reached the clinical trials until now but fortunately, research studies are still ongoing on this compound and its derivatives as KSP inhibitors [7].

In 2021, Fukai *et al.* reported the design and evaluation of a new prodrug, a S-trityl-L cysteine derivative targeting the KSP in cancer cells [20]. The new prodrug design was based on structural modification of the amino acid moiety of the STLC compound (Figure 3) in order to mask the KSP the activity of the STLC derivative through mimicking the structure of glutathione (GSH) until converted by the g-glutamyltransferase (GGT) to the active compound just near the tumor cells [20].

#### Dihydropyrrole derivatives

MK-0731 is a 2,4-diaryl-2,5-dihydropyrrole KSP inhibitor discovered in 2008 by Merck Sharp & Dohme^®^ (KSP ATPase IC_50_ = 2.2 nM) [2,12]. This compound is the only KSP inhibitor that reached the clinical trials from this group (ClinicalTrials. gov, NCT00104364) [12]. MK-0731 was tested to treat patients with advanced solid tumors such as non-small-cell lung cancer, cervical and ovarian cancer. Unfortunately, no clear outcomes were reported and the study was considered completed at the end of phase I with no complete remission or partial response results on tested patients [12].

#### Quinazolinone derivative

Ispinesib (SB-715992, CK0238273) was discovered in 2002 by Cytokinetics^®^ and GlaxoSmithKline^®^ as a KSP motor domain targeted inhibitor (Table 1, compound 1) [21]. Ispinesib was derived through chemical optimization of a series of compounds discovered by high-throughput screening drug discovery program [22,23].

**Table 1.** KSP inhibitors that reached clinical trials.

No.^†^	Structure	Inhibitor chemical class/company/ publication Year	Clinical trials (n)	Conditions
1.	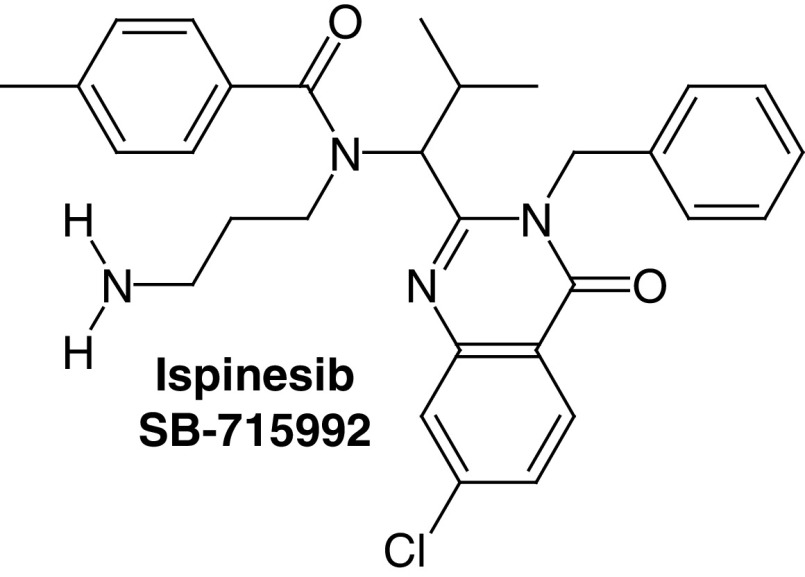	Quinazolinone Cytokinetics^®^ 2002	16 Clinical trials Phase I/II 14 completed and 2 terminated	Mono and combination therapy in various kinds of cancer diseases such as renal cell cancer (NCT00354250)^‡^, breast cancer (NCT00089973), head and neck cancer (NCT00095628), ovarian cancer (NCT00097409), prostate cancer (NCT00096499), non-small-cell lung cancer (NCT00085813), melanoma (skin) (NCT00095953), liver cancer (NCT00095992), metastatic colorectal cancer (NCT00103311)
**2.**	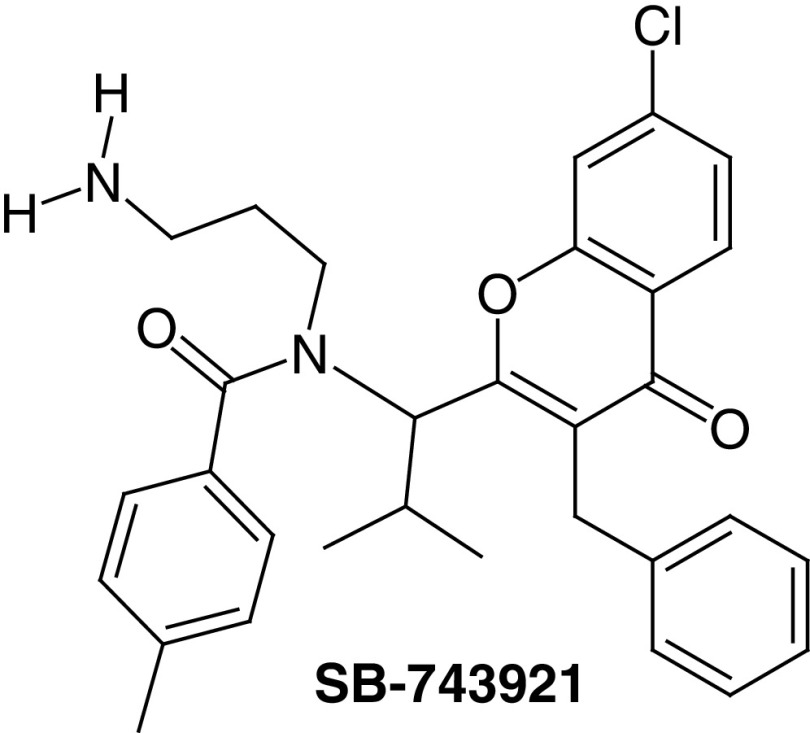	Chromen-4-one Merck^®^‘ 2006	Two clinical trials Phase I/II completed Both completed	Cholangiocarcinoma, solid tumors and lymphomas
**3.**	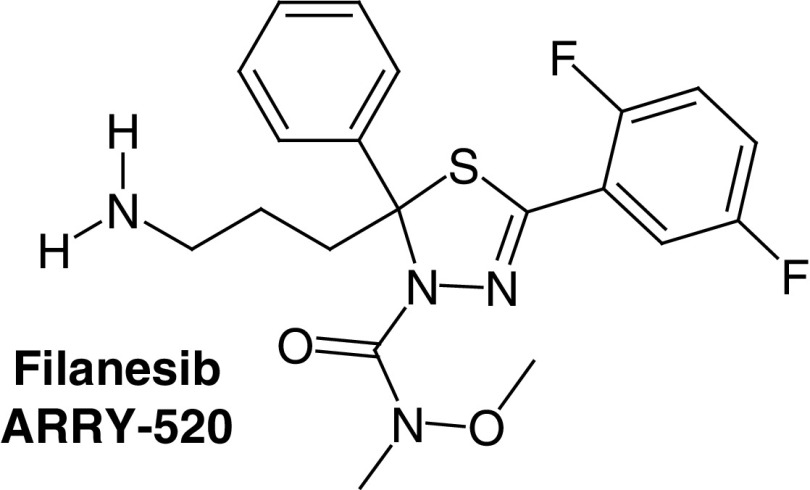	Thiadiazole Array Pharmaceuticals^®^ 2009	8 Clinical trials Phase I/II completed 1 active and 7 completed	Advanced myeloid leukemia, myeloma and advanced solid tumor
**4.**	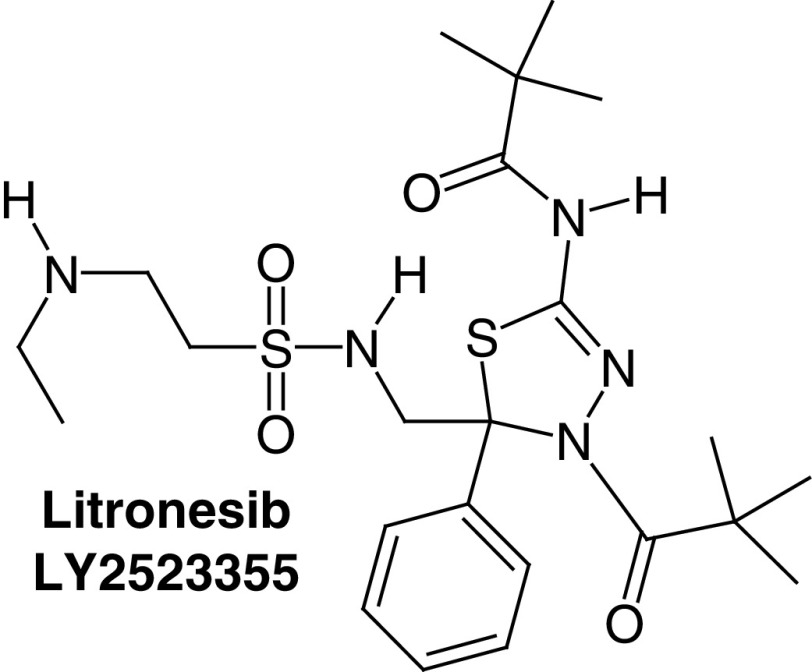	Thiadiazole Kyowa Kirin and Eli Lilly^®^ 2007	7 Clinical trials 6 completed and 1 terminated	Solid tumors, ovarian cancer, gastric cancer, prostate cancer and acute leukaemia

**NO.**	**Structure**	**Inhibitor chemical class/company/ publication year**	**Clinical trials** (n)	**Conditions**
**5**	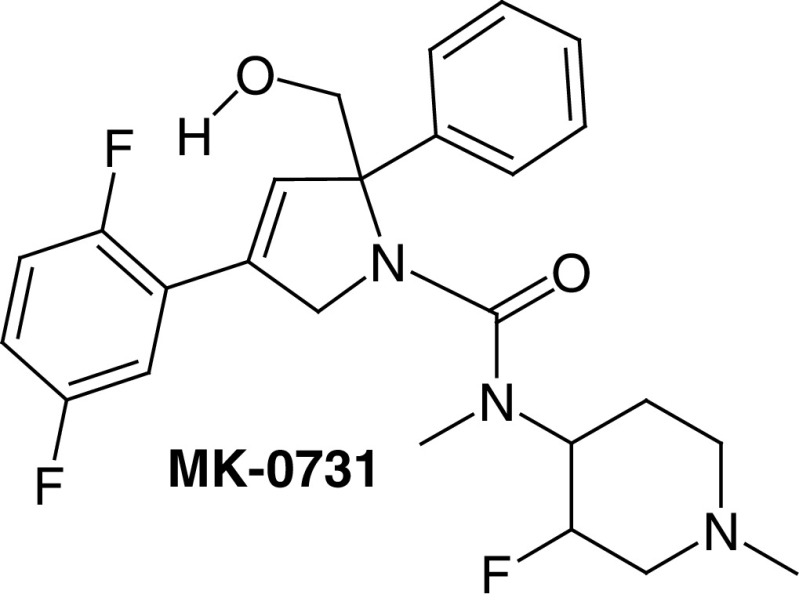	Pyrrole Merck Sharp & Dohme^®^ 2008	Phase I completed with stable disease response in treating non-small-cell lung cancer	Solid tumors such as lung, cervical and ovarian cancer
**6.**	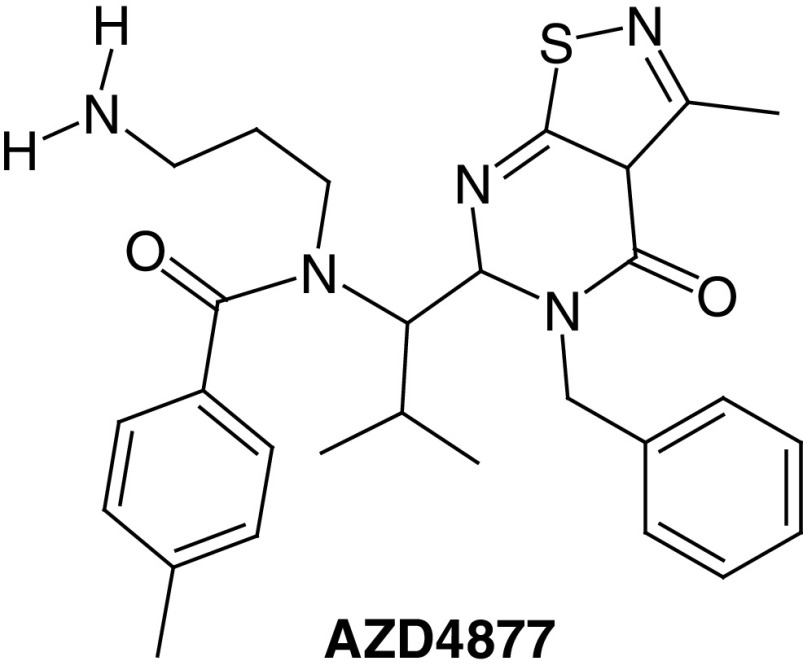	Thiazolopyrimidine AstraZeneca^®^ 2011	6 phase I/II clinical trials 3 Completed 3 Terminated	Solid cancers, acute myelogenous leukemia, non-Hodgkin lymphoma
**7.**	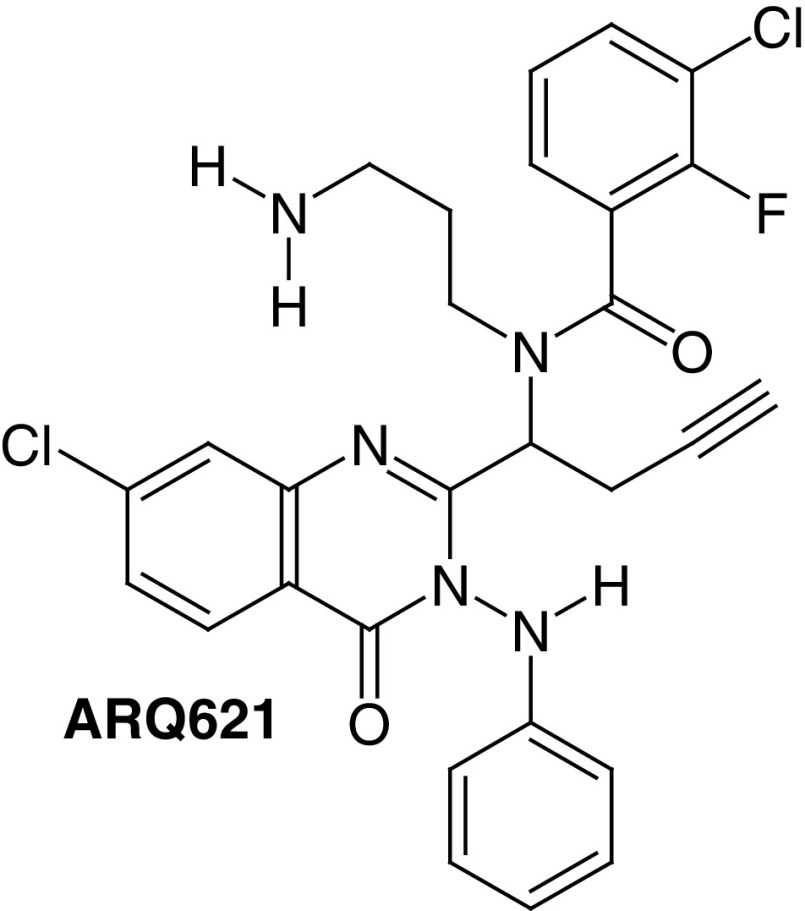	Quinazolinone ArQule^®^ 2008	One clinical study Halted after phase I	Hematological malignancies and metastatic solid tumors
**8.**	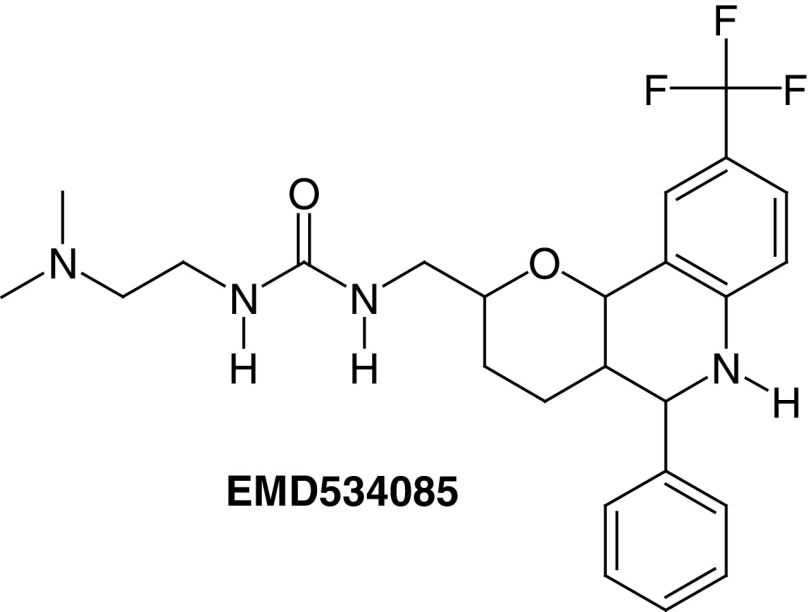	Pyrano[3,2-c]quinolones Merck-KGaA^®^ 2010	One clinical study	Refractory solid tumors, Hodgkin's lymphoma or non-Hodgkin's lymphoma

† Data from www.clinicaltrials.gov.

‡ Clinical trial identifier.

Crystallographic studies showed that ispinesib binds to the same binding site of monastrol almost 12 Å away from the ATP binding pocket. Eventually, binding of ispinesib to this binding pocket leads to locking the motor function of the KSP protein at the ADP state and preventing energy release, which leads to mitotic arrest and apoptotic cell death [4].

Later on, ispinesib was the first potent and specific inhibitor of KSP to go through clinical trials and to be tested for human disease. The KSP ATPase IC_50_ activity of this drug was less than 10 nM and it had a very well-accepted safety profile [12].

According to the US National Institute of Health (NIH) ispinesib was tested in 13 phase I/II clinical trials as monotherapy against several cancer diseases such as recurrent renal cell cancer, breast cancer, recurrent or metastatic head and neck cancer and liver and colorectal cancer. Although ispinesib showed a marginal safety profile but the efficacy of this compound as a single treatment is questionable and the best response was a partial response for ovarian and breast cancers [12,24].

As combination therapy, ispinesib went through three clinical trials in patients with solid tumors. The first one was with docetaxil (clinical trial no.: NCT00169520). In the second trial, ispinesib was combined with capecitabine (clinical trial number NCT00119171) and in the third one ispinesib was combined with carboplatini (clinical trial no.: NCT0011136578), the best results among these three combinations was a steady state response with capecitabine (an antimetabolite) and carboplatin [25–27].

An overall 16 clinical trials were performed on ispinesib, 14 were completed and two were terminated and none of these clinical trials resulted in a conclusive evidence of benefit [12].

ARQ 621 is another quinazolinone derivative that has the chemical structure of: N-(3-amino-propyl)-3-chloro-N-[1-(7-chloro-4-oxo-3-phenylamino-3,4-dihydro-quinazolin-2-yl)-but-3-ynyl]-2-fluoro-benzamide (Table 1, compound 7). ARQ 621 was originated first by ArQule^®^ in 2008 and later on, was developed by Merck & Co^®^ as an antineoplastic KSP inhibitor. The earliest *in vitro* studies showed that ARQ 621 displayed anticancer activity against a broad-spectrum human cancer cell lines [28]. ARQ 621 was inspected clinically in phase I clinical trials against hematological malignancies and metastatic solid tumors. Unfortunately, no clear responses were registered and no further development was reported for ARQ 621 [12,29].

#### Chromen-4-one derivative

##### SB-743921

After the discovery of ispinesib scientists performed lead optimization studies on this compound by replacing its quinazolinone core with a variety of heterocyclic and carbocyclic ring systems. Consequently, SB-743921 was discovered in 2006 by Merck^®^ through the isoteric replacement of the quinazoline ring in ispinesib with the chromen-4-one ring. SB-743921 (Table 1, compound 2) exhibited an ATPase IC_50_ activity of 0.1 nM, which is considered as a five-fold increase in potency against KSP over ispinesib [12].

SB-743921, was evaluated in phase I/II clinical trials against several malignancies. For example, phase I clinical study was conducted on SB-743921 in 2011 to patients with solid tumors (NCS001365513) the best result observed was as a partial response in patients suffering from cholangiocarcinoma. Another phase I/II clinical study was conducted on SB-743921 in 2014 on patients with Hodgkin's and non-Hodgkin's lymphoma. The best response was a partial response in patients with non-Hodgkin lymphoma.

A very promising clinical study on SB-715992 in treating patients with metastatic or recurrent head and neck squamous cell carcinoma is being conducted (ClinicalTrials.gov identifier: NCT00095628). In this study, patients received SB-715992 intravenously over 1 h on day 1 then therapeutic courses were repeated every 21 days with no signs of disease progression or un-tolerated toxicity were observed.

#### Thiadiazole derivatives

Phenotype-based screening as well as the isosteric replacement studies on ispinesib continued and led to the discovery of the Thiadiazole group as a new group of KSP inhibitors. The first compound of this group was K858 (KSP ATPase *in vitro* IC_50_ of 1.3 μM) [30,31]. K858 was observed to diminish the viability of human breast cancer cells such as MCF7 and SKBR3 along with human glioblastoma cells [32,33]. Yet, this compound did not reach the clinical stage though it served as a template to find more promising and potent thiadiazole-based KSP inhibitors such as filanesib (Arry-520) and litronesib (LY2523355) which showed promising results in clinical trials as KSP inhibitors.

Filanesib (Arry-520) (Table 1, compound 3) was developed by Array BioPharma^®^ in 2009. Filanesib is a thiadiazole derivative that has a prolonged cellular mitotic inhibition with an *in vivo* potency of 0.4–3.1 nM and *in vitro* ATPase IC_50_ of 6 nM. The chemical name of filanesib is ((2S)-2-(3-aminopropyl)-5-(2,5-difluorophenyl)-N-methoxy-N-methyl-2-phenyl-1,3,4-thiadiazole-3(2H)-carboxamide trifuoroacetate [31].

Eight clinical trials were performed for filanesib in patients with multiple myeloma, advanced/refractory myeloid leukemia and advanced solid tumors. Filanesib seems to be most promising KSP inhibitor as anticancer agent in the future.

The best response for filanesib as monotherapy in clinical trials was a partial response for multiple myeloma but neutropenia was the main serious drug-related toxicity. Fortunately, with combination therapy, better results were attained in clinical studies, best response was complete remission in phase I/II for myeloma when using the combination of pomalidomide, bortezomib, dexamethasone and the granulocyte colony-stimulating factor (G-CSF) filgrastim. Another combination therapy was also used with filanesib to treat multiple myeloma; this combination therapy includes bortezomib, pomalidomide, dexamethasone, filgraastim and carfilzomib and the results were promising [12]. Considering the promising clinical results of filanesib combination therapy against myeloma it will most likely enter phase III clinical trial [12].

Litronesib (LY2523355) (Table 1, compound 4) is another thiadiazole KSP inhibitor that has been discovered by Kyowa Kirin and Eli Lilly and Company^®^ in 2007, its chemical name is N-[(5R)-4-(2,2-dimethylpropanoyl)-5-[[2-(ethylamino)ethylsulfonylamino] methyl]-5-phenyl-1,3,4-thiadiazol-2-yl]-2,2-dimethylpropanamide. Its *in vitro* KSP ATPase IC_50_ value is 26 nM.

As shown by the file that was submitted by Eli Lilly and Company, litronesib was reported to inhibit the growth of 68 cancer cell lines [12,34,35]. Seven clinical trials were performed to evaluate the anticancer activity of litronesib in patients suffering from solid tumors, metastatic breast cancer, acute leukemia and small cell lung cancer. Litronesib is usually tested as combination therapy with a G-CSF drug such as filgrastim or pegfilgrastim to overcome neutropenia.

Litronesib alone or in combination showed partial remission as best response for small cell lung cancer and other kinds of cancers. At the end, six clinical trials were completed and one was terminated and so the Eli Lilly and Company decided to discontinue this drug [12].

#### Pyrrole derivatives

The best representative of the pyrrole KSP inhibitors is MK-0731 (Table 1, compound 5). MK-0731 showed potent and selective anticancer activity (inhibitory KSP ATPase IC_50_ = 2.2 nM) as reported by Cox *et al.* [7,36]. After completion of phase I clinical studies (ClinicalTrials. gov, NCT00104364) it has been shown that administration of MK-0731 as a 24-h infusion in patients with advanced solid malignancies causes disease stabilization for more than 5 months [12].

#### Thiazolopyrimidines

AZD4877 is another isostere to ispinesib that was developed in 2011 by AstraZeneca^®^ the core of AZD4877 is the thiazolopyrimidine heterocycle (Table 1, compound 6) AZD4877 displayed a KSP ATPase IC_50_ = 0.002 uM [37]. Six phase I/II clinical trials were performed on AZD4877 against several malignant conditions such as solid cancers, acute myelogenous leukemia and non-Hodgkin lymphoma. Unfortunately, the clinical results were not as expected and the development of AZD4877 has been stopped by AstraZeneca^®^ [12].

#### Hexahydro-2H-Pyrano[3,2]Quinolone

This group of compounds was first identified by high-throughput screening. Compound EMD534085 (Table 1, compound 8) showed KSP ATPase (IC_50_ = 8 nM). The lipophilic core of this compound improved its stability and pharmacokinetic profile [24]. The first clinical, phase I study (3 + 3 design) considered EMD 534085 safety, pharmacokinetics and as antineoplastic agent against solid tumors or lymphoma. Nevertheless, thus compound did not progress beyond the first phase of clinical trials [38].

### KSP ATP binding competitive inhibitors also named (helix-α4 & -α6 pocket) inhibitors

**Figure 4.**
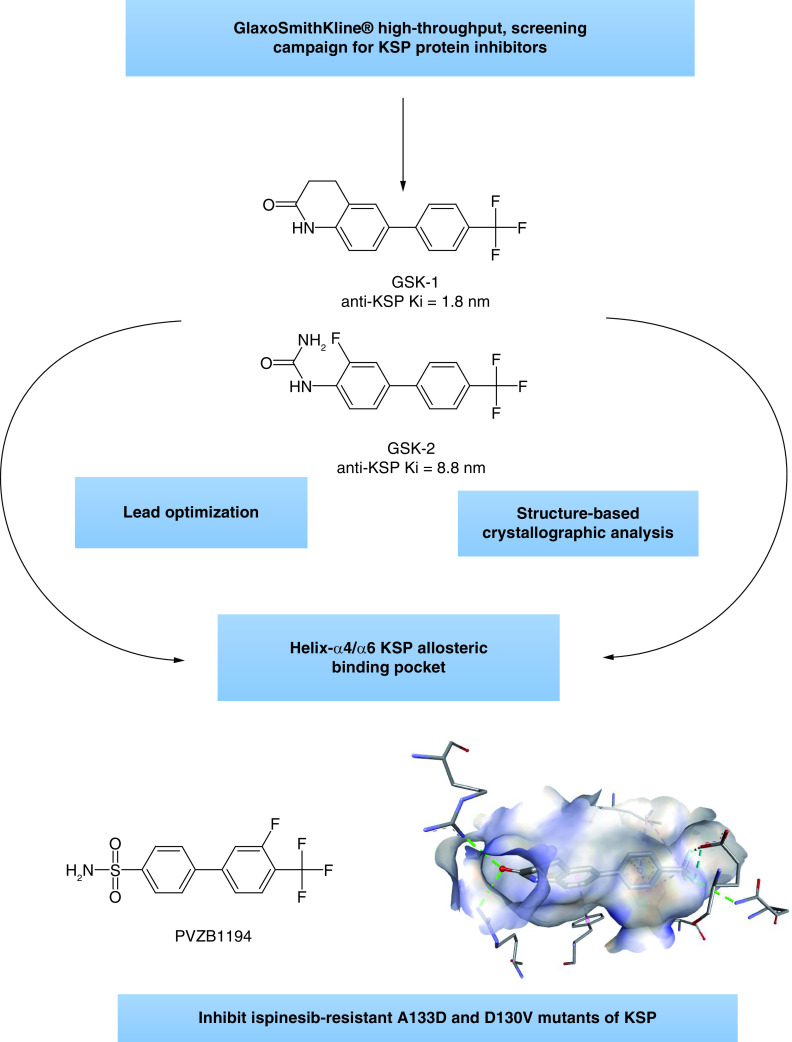
Biphenyl-based KSP inhibitors.

After an extensive high-throughput, screening campaign, the GlaxoSmithKline^®^ researchers discovered the bi-aryl (GSK-1 and GSK-2) KSP inhibitors in 2006. The anti-KSP Ki value of GSK-1 was 1.8 nM meanwhile the anti-KSP Ki of GSK-2 was 8.8 nM (Figure 4) [39].

The most distinctive remark regarding GSK-1 and GSK-2 as KSP inhibitors is that they were able to bind to ispinesib-resistant A133D and D130V mutants of KSP. This prompted the researchers to further analyze the binding mode of GSK-1 and GSK-2 inside the KSP motor domain using the structure-based crystallographic analysis and the cryoelectron microscopic technique [40]. Finally, It was confirmed that GSK-1 and GSK-2 bind between helix-α4 and helix-α6 pocket at the microtubule motor domain specifically through binding with the amino acid Leu295 as a site of labeling. The GSK-1 and GSK-2 allosteric mechanism of action is suggested to give the advantage of overcoming the cellular resistance to ispinesib as antineoplastic agent [39,41].

Unfortunately, GSK-1 and GSK-2 were not clinically successful and another series of modified biphenyl compounds, for example, PVZB1194 (Figure 4) are still under investigation as bi-phenyl KSP inhibitors [42].

## Challenges encountered by KSP inhibitors & novel therapeutic strategies to solve them

The main challenge that has been faced during the development of an active anti-KSP drug is the unsatisfactory efficacy results during advanced clinical trials [9]. So when compared with microtubule targeting drugs such as vinca alkaloids and taxanes, the KSP inhibitors still remain inferior in terms of efficacy and therapeutic benefit, especially when used as a monotherapy. This might be attributed to the fact that natural microtubule targeting drugs highly destabilize the microtubule-cytoskeleton system within the cell while the KSP inhibitors target an individual protein. Moreover, it is proposed that KSP inhibitors induce prolonged mitotic delay by disrupting the spindle assembly checkpoint which is expected to result in either cell death or what is named mitotic slippage (check point adaptation) through which the cell returns to the interphase and re-replicate [12,43].

On the other hand, the failure of the KSP inhibitors to clinically meet the researchers' expectations might also be attributed to the genomic instability due to incomplete mitotic inhibition which results in chromosomal segregation errors and genetic instability leading to fueling the malignancy and this explains the failure of KSP inhibitors in clinical trials when used as monotherapy [44]. Due to greater understanding of the molecular effect of KSP inhibitors during malignancies, scientists have found that KSP inhibitors' mechanism of action is highly restricted to M phase and to a lesser extent to G2 phase during cell cycle. Accordingly these drugs will only find their target in rapidly dividing tumor cells and this might also explain why these new drugs have failed during clinical trials [44].

Moreover, some other issues might be limiting the therapeutic success of KSP inhibitors such as the dose-limiting hematological adverse events. Most importantly, neutropenia, since the doubling time of the granulocyte precursors inside the human body is very short (63 h for promyelocytes and 17 h for the myeloblasts the KSP inhibitors are expected to cause reversible neutropenia. Although neutropenia is manageable through using the G-CSF support (pegfilgrastim) but still it is considered as the main dose limiting factor during the phase I dose escalation studies of KSP inhibitors.

KSP inhibitors might also suffer from drug due to low levels of inward cellular transporter or high levels of outward efflux transporter such as P-glycoprotein that can pump the anti-cancer drug outside the cell. Mutations at the ispinesib binding site were also identified in ispinesib-resistant HCT116 colorectal cells [45].

Novel therapeutic strategies are being investigated in order to overcome the previously mentioned challenges and to figure out why these KSP inhibitors failed to meet the expectations during clinical trials, the following are the most important ones:

### Combination therapy approach

There are two objectives for using combination therapy with KSP inhibitors; the first is to improve the clinical efficacy of the second-generation antimitotic inhibitors and the second is to maintain an acceptable range of overall toxicity. Therefore, it is believed that the use of combination therapy with KSP inhibitors during clinical trials would give better results [45]. Consequently, combination therapy seems to be a direct solution for ispinesib-resistant cancer strains [4,12]. Meanwhile, ispinesib combination therapy seems to be with better clinical results, especially when combined with cellular modulators such as Akt/Hsp70 signaling axis modulators or kinase inhibitors (Table 1) [10,11]. On the other hand, administrating filanesib (ARRY520) as combination therapy resulted in more promising results in phase I/II for multiple myeloma when used as combination with pomalidomide (anti-angiogenic), Bortezomib (proteasome inhibitor), Dexamethasone and the granulocyte colony-stimulating factor (G-CSF) filgrastim (please refer to Table 2 for the clinical trial results of filanesib in monotherapy combination therapy) [46,47].

**Table 2.** Results from clinical trials of Filanesib in monotherapy or in combination with other anti-myeloma agents in multiple myeloma patients.

Clinical trial identifier	Drug under study	Clinical phase	Drug regimen	Results
NCT00821249 Ref.	Filanesib with or without dexamethasone	Phase I monotherapy	Maximum tolerated dose of filanesib = 1.5 mg/m^2^ 14-day cycles	10% partial remission 3% minimal response 48% stable disease
		Phase II monotherapy	Filanesib iv 1.5 mg/m^2^ days 1, 2 14-day cycles	16% partial remission 6% minimal response 39% stable disease Progression-free survival: 1.6 months Overall survival: 19.0 months
		Phase II Combination with Dexamethasone	Filanesib iv 1.5 mg/m^2^ days 1, 2 Dexamethasone 40 mg oral weekly 14-day cycles	2% very good partial response 13% partial remission 6% minimal response 41% stable disease Progression-free survival: 2.8 months Overall survival: 10.7 months
NCT01248923 Ref.	Filanesib Bortezomib Dexametrhasone	Phase I combination therapy	Schedule 1 maximum tolerated dose: Filanesib = 1.5 mg/m^2^, days 1, 2, 15, 16 Bortezomib 1.3 mg/m^2^ days 1, 8, 15; Dexametrhasone 40 mg days 1, 8, 15 Schedule 2 maximum tolerated dose: Filanesib 3.0 mg/m^2^, days 1, 15 Bortezomib 1.3 mg/m^2^ days 1, 8, 15; Dexametrhasone 40 mg (days 1, 8, 15 28-day cycles	2% stringent complete response 9% very good partial response 9% partial remission 13% minimal response 62% stable disease Duration of response: 14.1 months
NCT01372540 Ref.	Filanesib Carfilzomib Dexametrhasone	Phase I Combination therapy	Part A maximum tolerated dose: Filanesib 1.5 mg/m^2^, days 1, 2, 15, 16 Carfilzomib 20/27 mg/m^2^, days 1, 2, 8, 9, 15, 16 Dexametrhasone 4 mg prior to Carfilzomib Part B maximum tolerated dose: Filanesib 1.5 mg/m^2^, days 1, 2, 15, 16 Carfilzomib iv 20/56 mg/m^2^ days 1, 2, 8, 9, 15, 16 Dexametrhasonem 40 mg days 1, 8, 15 28-day cycles	8% very good partial response 29% partial remission 13% minimal response PFS 4.8 months OS 24.9 months
NCT01989325 Ref.	Filanesib + carfilzomib	Phase II Combination therapy	Filanesib 1.25 mg/m^2^ iv, days 1, 2, 15, 16 Carfilzomib iv 20/27 mg/m^2^, days 1, 2, 8, 9, 15, 16 28-day cycles	7% very good partial response 21 % partial remission 5% minimal response 33% stable disease Progression-free survival: 8.5 months
NCT02384083 Ref.	Filanesib Pomalidomide Dexamethasone	Phase Ib Phase II	Filanesib iv 1.25 mg/m^2^, days 1, 2, 15, 16 Pomalidomide oral 4 mg, days 1–21 Dexamethasone oral 40 mg, days 1, 8, 15, 22 28-day cycle	12% very good partial response 54% partial remission 4% minimal response 23% stable disease Progression-free survival:7 months Overall survival 75% (at 24 months)

In the meantime, filanesib is considered as the only KSP inhibitor that showed anti-malignant activity in clinical trials, especially with multiple myeloma relapsed and refractory patients since Filanesib was able to promote the therapeutic results of standard treatments used in multiple myeloma patients such as proteasome inhibitors, dexamethasone and immunomodulating agents [46]. What is remarkable about filanesib treatment regimen is that the researchers have identified that patients with low alpha 1 acid glycoprotein (AAG) are more likely to benefit from filanesib and so the baseline levels of this biomarker can be used to identify patients who are more likely to achieve good therapeutic results from Filanesib [48].

### Gene therapy approach

The expression of the KSP genes can be regulated using the siRNA (short interfering RNA) strategy. Actually two anti-KSP siRNA formulations have reached the clinical trials the first one is ALN-VSP02 which was tested against endometrial cancer with metastases and showed a results of 2.7% complete remission [49]. Meanwhile, the second anti-KSP siRNA formulation entered clinical trials was 4SC-205 which was tested against multiple advanced malignancies but unfortunately the results of clinical studies against this formulation were not promising and only 28% of treated patients showed stable disease condition after completion of phase I [50].

Another gene-related strategy to target the KSP protein is directed against the kinesin superfamily (KIFs) which are a group of genes encoding proteins that control the microtubule-motility and function. Accordingly, there are 45 human KIF**s** discovered until now. *KIF11* is the main regulator of the KSP also known as Eg5 which is responsible for centrosomal separation and mitotic spindle reorganization [4]. It was found that *KIF11* is profoundly upregulated in many kinds of cancers such as glioblastoma and brain tumors [51].

On the other side, tripartite motif (TRIM) protein family are found to be a group of E3 ubiquitin ligase enzymes that are considered as an important coordinator of the cellular mitotic spindle system. Almost 80 TRIM human genes were identified [51]. Each TRIM protein has its specific interactor. In a research published by Venuto *et al.* in 2020, it was found that TRIM8 protein interacts with the mitotic spindle during centrosome separation [52]. Specifically, TRIM8 interacts with *KIFC1*, and *KIF11*/Eg5. Through this interaction, TRIM8 delays the mitotic progression and increases the chromosomal stability. This study provides future insights on the important role of TRIM8 in regulating the mitotic machinery through the interaction with KIF11. Inhibition of Trim8 binding site on the KSP enzyme or even blocking TRIM8 itself might be a convenient strategy to develop new and effective KSP inhibitors to treat brain cancer in TRIM8 related phenotypes [52].

Moreover, in 2014, Wang and Lin published a very important research article demonstrating the effect of knocking down the *KIF11* gene using two different shRNAs [53]. *KIF11* was successfully knocked down and western blot assays showed that knocking down technique was able to decrease *KIF11* expression by 58% [53].

In a related study also published in 2020 by Li *et al.*, it was found that knocking down the *KIF22* gene which is also a kinesin family gene that is related to colon cancer would inhibit colon cancer cell proliferation [54]. The question now would gene therapy be better than small molecule therapy? In fact, only one anti-KSP siRNA formulations has reached the clinical trials. ALN-VSP is made up of (80–100 nm in diameter) nanoparticle-based delivery system. ALN-VSP encloses two chemically modified siRNAs in a 1:1 molar ratio, these two different siRNAs targets both KSP and VEGFA, The phase I best response of ALN-VSP in trials (NCT01158079 and NCT00882180) was a complete remission of 2.7% in endometrial cancer with multiple liver metastases but unfortunately no patients were confirmed to have solely KSP mRNA knockdown [12,55] and so more clinical trials are still needed to confirm if gene therapy would be better than small molecule therapy or not.

### Targeted antimitotic KSP inhibitors (antibody–drug conjugates)

Antibody targeted therapy is a relatively novel technique that addresses directly the cancerous cells through antibody–drug conjugates. In a research article published in 2019 it was shown that a group of scientists from Novartis institute for biomedical research are working on a KSP inhibitor antibody–drug conjugates [56]. The aim of the study is to use the targeted therapy in order to improve the efficacy and tolerability profile of the already know SB715992 (ispinesib), and ARRY520 (filanesib) KSP inhibitors. The study has concluded that some antibody-filanesib conjugates have the potential for superior *in vivo* efficacy compared with ado-trastuzumab emtansine (Kadcyla^®^) which is an already FDA-approved HER2+ targeted cancer therapy.

At the end of the article, the researchers have indicated that further evaluations of these antibody–drug conjugates are currently ongoing. This study provides another future platform for designing more potent and selective KSP inhibitor-targeted anticancer agents in order to overcome the challenges that are facing the road toward finding an effective and tolerable KSP inhibitor drug [56].

### Half-sandwich metal complexes bearing the KSP inhibitor

Recently, in 2020 a group of bio-organometallic chemists published a research study that demonstrates the idea of synthesizing half-sandwich metal complexes bearing the KSP inhibitor ispinesib [57]. The aim of this combination is to make ispinesib more potent anticancer agent. The metals used in this study were Ruthenium (Ru), Osmium (Os), Rhodium (Rh) and Iridium (Ir). Markedly, the Ir and Rh ispinesib complexes revealed higher KSP inhibitory *in vitro* activity compared with the ispinesib activity. The metal KSP inhibitor complexes may be considered as a new strategy that may be a valuable future approach in order to increase the efficacy and promote further clinical trials for the promising KSP inhibitors [57].

### Designing dual inhibitors

Dual inhibitors are dual action drugs that possess dual inhibitory activity against two or more validated anticancer targets [58]. Dual inhibitors is emerging as a novel therapeutic strategy to combat cancer resistance. Recently, several research articles have discussed the idea of using the dual inhibitor strategy against KSP as anticancer target. Namely, CPUYL064 which is originally a KSP ATPase inhibitor that can induce cancer cells apoptosis [59] later on, CPUYL064 was modified by introducing several Aurora-A kinase inhibitors’ fragments [60]. The testing results showed that the resultant compounds were able to inhibit both the KSP protein and the Aurora enzyme. Some of the newly modified compounds were tested against the HepG2 cell line. The cytotoxic activity of the resultant compounds was prominent despite the moderate activities against the KSP protein and the Aurora-A kinase. For example, one of the compounds showed moderate anti-KSP (IC_50_ = 2.46 μM) and anti-Aurora-A kinase IC_50_ of 7.58 μM in addition that particular compound displayed a cytotoxic activity of (IC_50_ = 4.97 μM) against the HepG2 cell line. These results are considered promising for developing a novel class of dual inhibitors for cancer treatment [58].

## Conclusion

KSP is an attractive target for cancer treatment. The inhibitors of this protein are sub-divided into two main groups: 1-KSP (α2/loop L5/helix α3) inhibitors, 2-KSP (helix-α4 and -α6 pocket) inhibitors. Several KSP protein inhibitors have been studied, but only few have been tested in clinical trials for cancer treatment. monastrol was the first to be discovered by a phenotypic screening approach. Ispinesib is one of the most promising KSP inhibitors, it binds to the same binding site of monastrol almost 12 Å away from the ATP binding pocket but it suffers from cellular resistance. As a result, the GSK-1 and GSK-2 allosteric mechanism of action is suggested to give the advantage of overcoming the cellular resistance to ispinesib as antineoplastic agent. The most promising KSP inhibitor as anticancer agent in the future as a combination therapy is filanesib. In addition, novel therapeutic strategies are being approached in orde

## Future perspective

In this review, we intended to assess the clinical studies that have been performed on KSP inhibitors in the last decade and discuss the main challenges and possible future development of KSP inhibitors as anticancer therapeutics. Even though several phase I/II clinical trials evaluating the use of KSP inhibitors for the treatment of malignancies have now been completed ARRY520 (filanesib) is still the only KSP inhibitor that has shown some promising clinical results when used in combination with other anticancer drugs especially against hematological malignancies. Nevertheless, the unsatisfactory *in vivo* efficacy was the main factor that had limited the clinical success of most clinically tested KSP inhibitors. In summary, although the preclinical rationale for the efficacy of KSP inhibitors as anticancer agents was strong, never the less, the recent clinical trials results did not show any clear promising therapeutic results till now.

In this regard, there is still a continuous search for novel strategies to overcome the challenges that are facing the KSP inhibitors as therapeutic drugs. Hopefully, the new strategies will be able to show better clinical results but unfortunately the validity of KSP protein as anticancer target is being questionable especially in solid tumors. Meanwhile, the challenges that are encountering KSP inhibitors as therapeutic agents have to be further analyzed in order to find why these once highly appreciated antimitotic agents failed to clinically deliver their promise? Therefore, more investigational efforts have to be done to explain the anti-KSP inhibitors lack of efficacy and better understand how allosteric inhibitors interfere with the malignant cell cycle progression and the functions of mitotic spindles.

**Executive summary**

- Since the discovery of S-trityl-L cysteine (STLC) in 1992, many KSP (Eg5) inhibitors with various chemical scaffolds have been developed.
- KSP inhibitors are subdivided into two main groups the KSP α2/loop L5/helix α3 inhibitors and the KSP helix-α4 and -α6 pocket inhibitors.
- Ispinesib binds to the same binding site of monastrol almost 12 Å away from the ATP binding pocket.
- Filanesib seems to be most promising KSP inhibitor as anticancer agent in the future as a combination therapy.
- The GSK-1 and GSK-2 allosteric mechanism of action is suggested to give the advantage of overcoming the cellular resistance to ispinesib as antineoplastic agent.
- Novel therapeutic strategies are being approached in order to overcome the challenges that are facing the researchers who are developing new KSP inhibitors as anticancer drugs.
- More clinical trials are still needed to confirm if gene therapy would be better than small molecule therapy or not.
- The future of KSP inhibitors as cancer treatments is still full of challenges; but the new research strategies such as designing dual inhibitors, the antibody–drug conjugates (ADC), the combination therapy approach, and the gene therapy approach may carry a lot of solutions for these challenges.
